# The relevance of MRI‐based AI Predictive Modelling for Treatment's response sand personalized Clinical Trials design

**DOI:** 10.1002/alz70856_106596

**Published:** 2026-01-11

**Authors:** Luca Villa, Nicolas Guizard, Mathilde Borrot, Ayoub Gueddou, Audrey Gabelle, Elizabeth Gordon, Olivier Courreges

**Affiliations:** ^1^ Qynapse, Paris, Île‐de‐France, France; ^2^ Montpellier Excellence University, Montpellier, France

## Abstract

**Background:**

The heterogeneity of patient clinical progression is a key challenge in Alzheimer's disease (AD). This is especially the case when conducting disease‐modifying clinical trials in patients with mild cognitive impairment (MCI) and early AD where, historically, large proportions of patients in placebo groups show subtle cognitive decline within the period of the trials, hindering the ability to detect treatment effects. Recent advances in Artificial Intelligence (AI) have allowed rapid progress to be made in the field of predictive modeling, enabling the integration of clinical, demographic, genetic, and neuroimaging data to provide accurate personalized predictions of prognosis at an individual level. So far, these methods have remained in the domain of academic research but have huge potential if applied to the enrichment of real‐world clinical trials in AD, as well as in the prioritization of medication prescription for newly approved treatments.

**Method:**

We have developed QyScore®, an advanced fully‐automated CE‐Marked and FDA‐cleared medical imaging platform that is certified for grey and white matter segmentation, and was used to identify a reduction in grey matter atrophy associated with treatment in a recent AD clinical trial. Moreover, we have developed QyPredict®, an AI‐based predictive modelling platform designed to predict clinical progression in central nervous system diseases.

**Result:**

By integrating the neuroimaging quantifications generated by QyScore® into QyPredict®, alongside key demographic, clinical, and genetic data from a wide variety of patients and healthy controls, we have achieved high prediction accuracies when predicting clinical decline – with the following range of performance metrics, depending on clinical population and prediction target: Balanced Accuracy: 0.70‐0.81, Sensitivity: 0.73‐0.80, Specificity: 0.60‐0.88, Precision: 0.77‐0.81, Negative Predictive Value: 0.59‐0.87, F1: 0.74‐0.81.

**Conclusion:**

Given the ability of this predictive modelling platform to identify patients at the highest risk of clinical decline, it could be invaluable for the stratification of the right patient’ target. Moreover, its ability to predict patients with the greatest likelihood for remaining clinically stable could be invaluable for the identification and exclusion of patients in future clinical trials that are unlikely to show clinical decline – therefore improving the ability of future trials to detect treatment effects.

